# Analysis of 2-(2-Phenylethyl)chromones by UPLC-ESI-QTOF-MS and Multivariate Statistical Methods in Wild and Cultivated Agarwood

**DOI:** 10.3390/ijms17050771

**Published:** 2016-05-23

**Authors:** Yuanbin Li, Nan Sheng, Lingli Wang, Shijie Li, Jiannan Chen, Xiaoping Lai

**Affiliations:** 1 School of Chinese Materia Medica, Guangzhou University of Chinese Medicine, Guangzhou 510006, China; afanliyb@sina.com (Y.L.); shengnan@gzucm.edu.cn (N.S.); lisj666@163.com (S.L.); chenjiannan@gzucm.edu.cn (J.C.); lai_xp88@sina.com (X.L.); 2 Dongguan Mathematical Engineering Academy of Chinese Medicine, Guangzhou University of Chinese Medicine, Dongguan 523808, China

**Keywords:** *Aquilaria*, chromone, metabonomics, LC-MS, biomarker, CITES, conservation

## Abstract

Agarwood is the fragrant resinous material mainly formed from species of *Aquilaria*. 2-(2-phenylethyl)chromones, especially the highly oxidized 5,6,7,8-tetrahydro-2-(2-phenylethyl)chromones, are the main representative compounds from agarwood. It is important to determine whether agarwood in trade is from cultivated trees or natural trees in the Convention on the International Trade in Endangered Species (CITES). We characterized the 2-(2-phenylethyl)chromones in agarwood by ultra-performance liquid chromatography coupled with electrospray ionization mass spectrometry (UPLC–ESI-QTOF-MS) and differentiated wild from cultivated agarwood by metabolomic analysis. A total of 141 chromones including 50 potentially new compounds were evaluated as belonging to four structural classes (unoxidized 2-(2-phenylethyl)chromones, 5,6,7,8-tetrahydro-2-(2-phenylethyl)-chromones, bi-2-(2-phenylethyl)chromones, and tri-2-(2-phenylethyl)chromones). The metabolic difference between wild and cultivated agarwood was analyzed by component analysis (PCA) and orthogonal partial least squares discriminant analysis (OPLS-DA). Fourteen markers of metabolisms in wild and cultivated agarwood were constructed (e.g., 6,7-dimethoxy-2-(2-phenylethyl)chromone, 6,8-dihydroxy-2-(2-phenylethyl)chromone, 6-methoxy-2-(2-phenylethyl)chromone, *etc.*). These results indicated that UPLC–ESI-QTOF-MS-based metabonomics analysis in agarwood may be useful for distinguishing wild agarwood from cultivated agarwood.

## 1. Introduction

Agarwood, also known as aloeswood or eaglewood in several regions, *chen xiang* (China), *jinkoh* or *kanankoh* (Japan), *gaharu* or *kalabak* (Malaysia and Indonesia), *krissna* (Thailand and Lao), *agar* (India), and *oud* (Middle East), is a fragrant resinous heartwood obtained from certain trees belonging to Malvales Thymelaeaceae [1,2]. It is widely accepted that agarwood was created as a response of the tree to various forms of injury, including natural injuries and artificial injuries [3,4,5]. The supply of agarwood (wild sources) is far lower than the market demand because only a few source trees in nature can actually produce agarwood, and its production is slow. Due to the exploitation of this tree, all *Aquilaria* spp. were listed on Appendix II of the Convention on the International Trade in Endangered Species (CITES) in 2004. Nine *Aquilaria* species—*A. crassna* (critically endangered), *A. rostrata* (threatened), *A. banaensae*, *A. beccariana*, *A. cimingiana*, *A. hirta*, *A. malaccensis*, *A. microcarpa*, and *A. sinensis* (vulnerable)—were listed on the International Union for Conservation of Nature (IUCN) red list as endangered species [6]. It is important for CITES scientific and management authorities to determine whether agarwood in trade is from cultivated trees (legal) or natural trees (illegal). Technology needs be developed that can distinguish between wild and cultivated agarwood, which is one of the main problems in the trade of this good.

Agarwood contains a complex mixture of compounds including agarofurans, agarospiranes, guaianes, eudesmanes, eremophilanes, prezizaanes, 2-(2-phenylethyl)chromone derivatives, aromatics, triterpenes, and many others. It has been reported that 54 2-(2-phenylethyl)chromones have been isolated from various *Aquilaria* species [4,5]. In recent years, 35 new 2-(2-phenylethyl)chromones, including 13 5,6,7,8-tetrahydro-2-(2-phenylethyl)chromones, have been identified in differing qualities of agarwood products [7,8,9,10,11,12,13]. Among 89 2-(2-phenylethyl)chromones, 33 highly oxidized chromones appear to be unique to agarwood and are not found in healthy *Aquilaria* specimens [2]. Lancaster *et al.* evaluated the use of the 5,6,7,8-tetrahydro-2-(2-phenylethyl)-chromones (*m*/*z* 319.118 or 349.129) for agarwood identification and for commercial product verification imported into the USA [2]. Espinoza *et al.* distinguished wild from cultivated agarwood (*Aquilaria* spp.) using direct analysis in real time and time of-flightmass spectrometry (DART-TOFMS) [14].

In this study, an ultra-performance liquid chromatography coupled with electrospray ionization mass spectrometry (UPLC–ESI-QTOF-MS) method was developed for identifying different types of 2-(2-phenylethyl)chromones derivatives and differentiating between sources and screening metabolomic indicators for future metabolic studies. MS characterization of 2-(2-phenylethyl)chromone derivatives were summarized to identify 2-(2-phenylethyl)chromones, and component analysis (PCA) and orthogonal partial least squares discriminant analysis (OPLS-DA) was employed to compare the metabolic difference between wild and cultivated agarwood.

## 2. Results

### 2.1. Characterization of 2-(2-Phenylethyl)chromone Derivatives by UPLC-ESI-QTOF-MS

Accurate mass data were acquired in the full scan analysis, and product ion mass data were acquired via the IDA (information-dependent acquisition) method. A total of 141 chromones, distributed in four major classes (unoxidized 2-(2-phenylethyl)chromones, highly oxidized 5,6,7,8-tetrahydro-2-(2-phenylethyl)-chromones, bi-2-(2-phenylethyl)chromones, and tri-2-(2-phenylethyl)chromones) were analyzed in the present study. The major peaks, which were identified according to their elution order, are listed in Table 1 and Table 2. All of the compounds were identified by interpretation of their MS and MS/MS spectra and also by previously reported data in the literature.

#### 2.1.1. Identification of 2-(2-Phenylethyl)chromones (Unoxidized) in Agarwood

**Figure 1:**
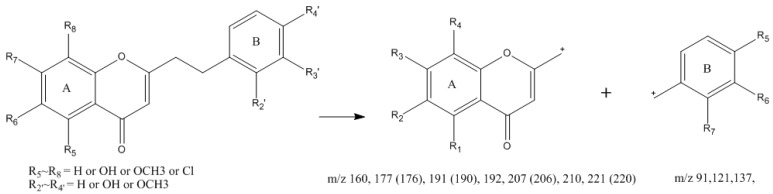
Characteristic fragments of 2-(2-phenylethyl)chromones.

It was reported that the most 2-(2-phenylethyl)chromones in agarwood have been substituted by methoxy or/and hydroxy groups. The characteristic fragmentation behaviors of the 2-(2-phenylethyl)chromones are the cleavages of the CH_2_–CH_2_ bond between chromone moiety and phenyl moiety. These compounds have two main types of fragment peaks, such as ions formed by different substituted chromone moieties (A) (*m*/*z* 160 [C_10_H_8_O_2_], 177 [C_10_H_8_O_2_ + OH], 191 [C_10_H_8_O_2_ + OCH_3_], 192 [C_10_H_6_O_2_ + OH × 2], 210 [C_10_H_6_O_2_ + Cl + OH], 221 [C_10_H_7_O_2_ + OCH_3_ × 2]), while ions formed by different substituted benzyl moieties (B) (*m*/*z* 91 [C_7_H_7_], 107 [C_7_H_6_ + OH], 121 [C_7_H_6_ + OCH_3_], 137 [C_7_H_5_ + OH + OCH_3_]) (Figure 1). Based on the accurate mass and characteristic ions, 60 2-(2-Phenylethyl)chromones were tentatively identified in Agarwood.

Peak 53 with *m*/*z* 251 was tentatively identified as 2-(2-phenylethyl)chromone. Its MS/MS data displayed the fragment ions at *m*/*z* 160 and 91, corresponding to benzyl ion and chromone ions [15].

Peaks 20, 27, 28, 32, 35, and 42, showed a similar molecular ion at *m*/*z* 267. Peaks 20, 27, and 35 were identified as qinanone D isomers [13], all of which are substituted by hydroxyl in the benzyl moieties, although it was not possible to distinguish the position of hydroxyl because they showed the same fragmentation pattern. Peaks 28, 32, and 42 were identified as 7-hydroxy-2-(2-phenylethyl)chromone isomers, all of which are substituted by hydroxyl in the chromone moieties [15].

2-[2-(4-methoxyphenyl)ethyl]chromone (Peak 52) was proposed by elucidating and comparing their MS data in the literature [13]. Peaks 54 and 58, with the same fragment ion at *m*/*z* 190, were detected as 6-methoxy-2-(2-phenylethyl)chromone and its isomer [16].

Eleven isomers (Peaks 1, 3, 4, 6, 9, 13, 17, 19, 25, 46, 51) displayed similar molecular ions at *m*/*z* 283 and the same molecular formula C_17_H_14_O_4_. The fragmentation ions at *m*/*z* 177 and 107 were observed in compounds 3, 4, 6, 9, and 19, while the fragmentation ions at *m*/*z* 192 and 91 were detected in compounds 1, 13, 17, 25, 46, and 51. Based on the 2-(2-phenylethyl)chromones isolated from agarwood in a previous study, these compounds were tentatively identified as 6-hydroxy-2-[2-(4-hydroxyphenyl)ethyl]chromone isomers and 6,8-dihydroxy-2-(2-phenylethyl)chromone isomers, respectively [15]. Seven of these compounds were detected in agarwood for the first time.

Peak 39 had the molecular formula C_18_H_16_O_4_ and showed two fragment ions at *m*/*z* 121 and 176 corresponding to C_7_H_6_ + OCH_3_ and C_10_H_7_O_2_ + OH, which resulted from the breaking of CH_2_–CH_2_ bond [17]. Peaks 29, 31, and 44, a MS/MS spectrum with the fragment ions at *m*/*z* 191 and 107, were tentatively considered to be 6-methoxy-2-[2-(4-hydroxyphenyl)ethyl]chromone isomers [11], while peaks 18, 34, 36, and 60, the fragment ions at *m*/*z* 206 and 91, were proposed as 6-hydroxy-7-methoxy-2-(2-phenylethyl)chromone isomers [15].

Peak 56 (C_17_H_13_ClO_3_), the ratio of 33.3% observed from the ^37^Cl/^35^Cl (303.0598/301.0626), whose MS^2^ ions at 210 (C_10_H_6_O_2_ + Cl + OH) and 91 (C_7_H_7_), was assigned to the 8-chloro-6-hydroxy-2-(2-phenethyl)chromone [10].

Peaks 50 and 57, with *m*/*z* 311, were tentatively identified as 6,7-dimethoxy-2-(2-phenylethyl) chromone and 6-methoxy-2-[2-(4-methoxyphenyl)ethyl]chromone, according to their MS data and information previously reported [4].

Nine isomers (peaks 7, 12, 14, 15, 24, 41, 45, 47, and 49) showed the similar molecular ion at *m*/*z* 313 (C_18_H_16_O_5_). Based on the substituted position, these compounds can be divided into four groups. Peaks 7, 41, and 47, the chromone moiety (A) substituted by OH, OCH_3_ and the phenyl moiety substituted by OH, were characterized as 6-hydroxy-7-methoxy-2-[2-(4’-hydroxyphenyl)ethyl]chromone isomers [7]. Peaks 12 and 14 were tentatively suggested to be 6-hydroxy-2-[2-(4’-hydroxy-3’-methoxy phenyl)ethyl]chromone and its isomer [11]. Peaks 15, 24, and 45 (A ring substituted by two hydroxys, B ring substituted by methoxy) presented the same MS/MS ion at 121 and were tentatively characterized as 6,7-dihydroxy-2-[2-(4’-methoxyphenyl)ethyl]chromone isomers [7]. Peak 49 showed the MS/MS ions at 222, 207, 178, and 91, were tentatively characterized as 6,8-dihydroxy-7-methoxy-2-(2-phenylethyl)chromone.

Eight isomers (peaks 16, 21, 23, 30, 33, 37, 40, and 59) displayed the same molecular formula C_19_H_18_O_5_. Based on the molecular formula provided for their accurate masses and the previously reported data, Peaks 16, 21, and 30, were characterized as Qinanone G isomers [12], and peaks 40 and 59 as 7-hydroxy-6-methoxy-2-(4-methoxyphenethyl)chromone isomers [8], although it was not possible to distinguish between them because they showed the same fragmentation pattern. Peaks 33 and 37 were identified as 6-methoxy- 2-[2-(4-hydroxy-3-methoxyphenyl)ethyl]chromone isomers, and both of these compounds displayed MS/MS ions at *m*/*z* 191 and 137. Peak 23 was tentatively characterized as 8-hydroxy-6,7-dimethoxy-2-(2-phenethyl)chromone by comparing its MS data with those previously reported [18].

Peaks 2 and 5 were found to be 6,8-dihydroxy-2-[2-(3′-hydroxy-4′-methoxyphenyl)ethyl]chromone isomers. Their MS/MS spectrum gave fragments corresponding to the benzyl moiety at *m*/*z* 137 [7].

Peak 55, with a [M + H]^+^ ion at *m*/*z* 331 (C_18_H_15_ClO_4_), was tentatively identified as 8-chloro-6-hydroxy-2-(4-methoxyphenethyl)chromone according to its accurate masses and the reported data [10]. Peak 48 was assigned as 6,7-dimethoxy-2-[2-(4-methoxyphenyl)ethyl]chromone [4].

Five isomers (peaks 8, 10, 11, 38 and 43) displayed similar molecular ions at *m*/*z* 283 and the same molecular formula C_19_H_18_O_6_. According to its MS data (206 and 137), they had been tentatively proposed to be 6-hydroxy-7-methoxy-2-(4-hydroxy-3-methoxyphenethyl)chromone isomers [7]. These compounds showed a structure based on the 2-(2-phenylethyl)chromone skeleton.

Peak 22 and 26 had been tentatively assigned to 6,7-dimethoxy-2-[2-(3′-hydroxy-4′-methoxyphenyl)ethyl]chromone isomers. These compounds, with the molecular ion at *m*/*z* 357, displayed MS/MS ions at *m*/*z* 220 and 137 [7].

#### 2.1.2. Identification of 5,6,7,8-Tetrahydro-2-(2-phenylethyl)chromones in Agarwood

**Figure 2:**
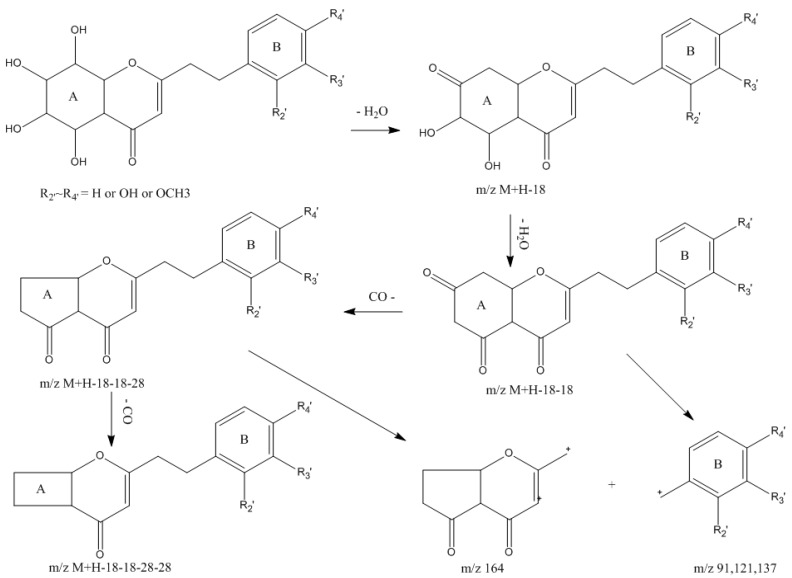
Proposed main fragmentation pathway of 5,6,7,8-tetrahydro-2-(2-phenylethyl)chromones.

The MS fragmentation behaviors of 5,6,7,8-tetrahydro-2-(2-phenylethyl)chromones were characterized as the successive neutral losses of two H_2_O and two CO molecules, and the cleavages of the CH_2_–CH_2_ bond from the molecular ions and fragment ions (Figure 2). In the present work, 27 5,6,7,8-tetrahydro-2-(2-phenylethyl)chromones were tentatively identified in agarwood.

Peak 66 was identified as agarotetrol [19]. The MS/MS of this compound displayed the fragment ions at *m*/*z* 301, 283, 255, and 227, in accordance with the successive losses of waters (301, 283) as well as subsequent two CO (255, 227). Fragment ion at *m/z* 164 corresponded to the loss of C_7_H_7_ (91) from the fragment ion of 255. Additionally, another agarotetrol isomer was detected in the extract (compound 73) that matched with aquilarone B [19].

Peak 79 gave a molecular ion at *m*/*z* 287 that was tentatively determined as 6,7-dihydroxy-5,6,7,8-tetrahydro-2-(2-phenylethyl)chromone [16]. Peaks 73, 76, 77, and 80 showed the molecular ion at *m*/*z* 303 (C_17_H_18_O_5_) and were identified as 5,6,7-trihydroxy-5,6,7,8-tetrahydro-2-(2-phenylethyl)chromone isomers. The MS/MS spectrum with the fragments ions at *m*/*z* 285, 267, 239, and 211 was in accordance with the successive neutral losses of two H_2_O molecules ([M + H − 18 − 18]^+^) and then two CO molecules ([M + H − 18 − 18 − 28 − 28]^+^), but the data presented by MS/MS were impossible to distinguish between these stereoisomers [8]. Three of them were characterized here for the first time in agarwood. Peaks 72 and 75 gave a molecular ion at *m*/*z* 333 (303 + OCH_2_) that was tentatively determined as 5,6,7-trihydroxy-8-methoxy-5,6,7,8-tetrahydro-2-(2-phenylethyl)chromone isomers [20]. Peaks 74 and 78, with the molecular formula C_18_H_20_O_6_, yielded the fragments at *m*/*z* 315 and 121. Where the product ion (*m*/*z* 315) resulted from the loss of H_2_O, the fragments at *m*/*z* 121 matched the methoxylphenyl ion (121). These compounds were tentatively characterized as 5,6,7-trihydroxy-2-(4-methoxyphenethyl)-5,6,7,8-tetrahydrochromone isomers [8].

Peaks 62 and 63 at *m*/*z* 335 (C_17_H_18_O_7_) were assigned to aquilarone F isomers by comparing molecular ion and fragments ions of agarotetrol (319 + 16), while peaks 67 and 71 were tentatively attributed to 5,6,7,8-tetrahydroxy-2-(4-methoxyphenethyl)-5,6,7,8-tetrahydrochromone [11,21]. Peak 61 at *m*/*z* 365 (319 + O + OCH_2_), based on the agarotetrol skeleton, was tentatively characterized as aquilarone A [11].

Six isomers (peaks 64, 65, 68, 70, 81 and 85) showed the molecular ion at *m*/*z* 301. Based on the molecular formula provided for their accurate masses and the reported data, they were identified as 2,3-dihydroxy-5-phenethyl-2,3-dihydro-1aH-oxireno[2,3-f]chromen-7(7bH)-one isomers, although it was not possible to distinguish between them because they showed the same fragmentation pattern [8]. The MS/MS of these compounds showed fragments at *m*/*z* 283, 255, 227, and 91 corresponding to [(M + H) − 18]^+^ (283) and the successive losses of CO (255, 227) from the main fragment (283), as well as the phenyl ions (91). Five of them were potentially new compounds.

Peak 82, 84, and 87 displayed a molecular ion at *m*/*z* 337 (C_17_H_17_ClO_5_). The QTOF analysis presented MS/MS fragments at *m*/*z* 319, 301, 283, 265, 192, and 91. The fragment ions at *m*/*z* 319, 301, 283, and 265, corresponded to the successive losses of two H_2_O and two CO molecules. In addition, the *m*/*z* ions at 192 and 91 resulted from the breakage of the CH_2_–CH_2_ bond from the fragment ion of 283. Based on these data, Peaks 82, 84, and 87 were proposed as 8-chloro-5,6,7-trihydroxy-2-(2-phenylethyl)-5,6,7,8-tetrahydrochromone and its isomers [16]. Two of them were detected for the first time in the *Aquilaria* species.

The molecular formula C_18_H_19_ClO_6_ was assigned to compound 83 and 86. These compounds were tentatively identified as 8-chloro-5,6,7-trihydroxy-2-(4-methoxyphenethyl)-5,6,7,8-tetrahydrochromene and its isomers [8], one of which was found in the extracts of agarwood for the first time.

#### 2.1.3. Identification of Bi-2-(2-phenylethyl)chromones in Agarwood

**Figure 3:**
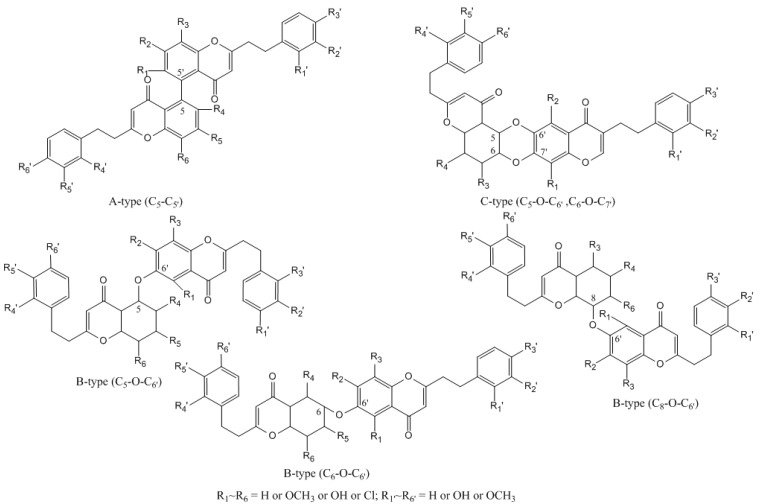
The main structures of bi-2-(2-phenylethyl)chromones (A-type, B-type, and C-type).

Bi-2-(2-phenylethyl)chromones represent another important role of metabolites characterized in this paper, only 7 of which had previously been detected in agarwood, 44 of which were potentially new chromones. These compounds have three main types of structure skeleton: A-type bi-2-(2-phenylethyl)chromones (two unoxidized 2-(2-phenylethyl)chromone units linked as C_5_ → C_5’_), B-type bi-2-(2-phenylethyl)chromones (an unoxidized 2-(2-phenylethyl)chromone and a 5,6,7,8-tetrahydro-2-(2-phenylethyl)chromone linked as a C_5_–O–C_6’_, C_8_–O–C_6’_, or C_6_–O–C_6’_ bond), and C-type bi-2-(2-phenylethyl)chromones, which possess a dioxan ring, resulting in dehydration between the C_5_–O–C_6’_ and C_6_–O–C_7’_ positions (Figure 3).

Peaks 123, 130, 135, and 138 showed a similar molecular ion at *m*/*z* 531 [M + H]^+^, with the same molecular formula C_34_H_26_O_6_ as the positive ionization mode. Their MS/MS data provided the fragment ions at *m*/*z* 440 and 267, in accordance with the loss of the methylphenyl ion and the breakage of the C_5_–C_5’_ bond; thus, these compounds have been assigned to be 6,8′-dihydroxy-2,2′-diphenethyl-4H,4′H-[5,5′-bichromene]-4,4′-dione (AH11) isomers (A-type) [22]. Only one of them had been reported in agarwood.

Six isomers (peaks 105, 112, 126, 129, 134, and 137) were tentatively characterized as 6,7,8′-trihydroxy-2,2′-diphenethyl-4H,4′H-[5,5′-bichromene]-4,4′-dione isomers (hydroxy AH11). These compounds at *m*/*z* 547 displayed MS/MS ions at *m*/*z* 529, 456, 282, 267, and 91. The fragment ions at *m*/*z* 282 and 267 resulted from the bihydroxy-2-(2-phenylethyl)chromone (282) and hydroxy-2-(2-phenylethyl)chromone (267), respectively. Peaks 122, 124, and 136, with [M + H]^+^ at *m*/*z* 561 (C_35_H_28_O_7_), were 30 Da (CH_2_O) heavier than that of AH11 (531). The MS/MS analysis of these compounds yielded the fragment ions at *m*/*z* 470 and 121, corresponding to [(M + H) − 91]^+^ and C_7_H_6_ + OCH_3_ (121), respectively. Thus, they were identified as methoxy AH11 isomers. These nine compounds were potentially new compounds.

**Figure 4:**
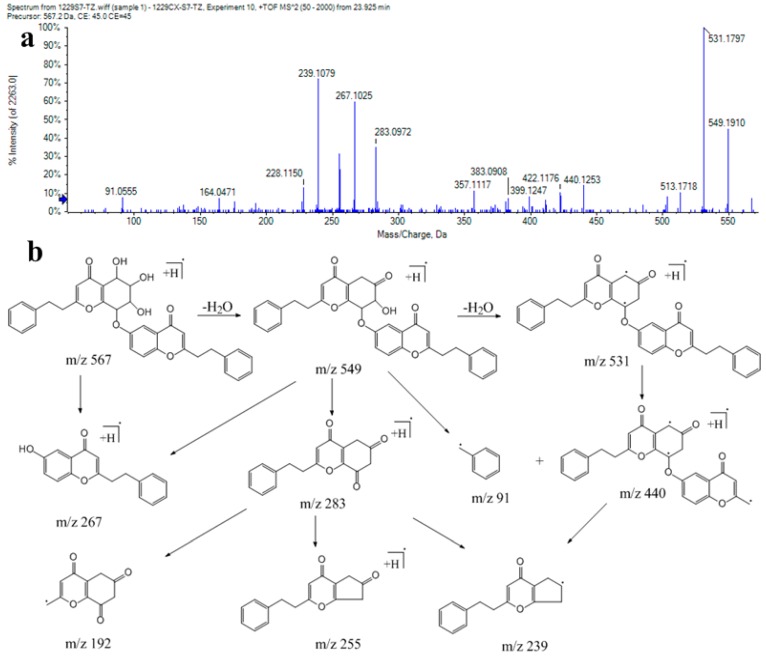
(**a**) MS/MS spectrum of compound 117; (**b**) proposed fragmentation pathway of compound 117.

Peaks 91, 96, 99, 108, 113, and 117 (*m*/*z* 567) were tentatively identified as 2-phenethyl-6-(((5*S*,6*R*,7*R*,8*S*)-5,6,7-trihydroxy-4-oxo-2-phenethyl-5,6,7,8-tetrahydro-4H-chromen-8-yl)oxy)chromone (AH13) isomers [22]. The MS/MS analysis of this compound yielded the fragment ions at *m/z* 549, 531, 513, 283, 255, 192, and 91 (Figure 4).

Peak 133, the derivative of bi-2-(2-phenylethyl)chromone (B-type), gave the molecular ion at *m*/*z* 585, with the molecular formula C_34_H_29_O_7_Cl. Its MS/MS spectrum gave fragments at *m*/*z* 319 and 267 in accordance with the breakage of the molecular ion. This compound was tentatively determined as 6-((8-chloro-6,7-dihydroxy-4-oxo-2-phenethyl-5,6,7,8-tetrahydro-4H-chromen-5-yl)oxy)-2-phenethylchromone, and was found in the extract of *Aquilaria* spp. for the first time.

Thirteen isomers (Peaks 88, 89, 90, 92, 95, 97, 101, 104, 106, 111, 114, 118, and 119), with a [M + H]^+^ at *m*/*z* 583, showed MS/MS fragment ions at *m*/*z* 565, 547, 519, 192, and 91, in accordance with the successive losses of two water molecules and a CO molecule [M + H − 2H_2_O − CO]^+^ and the breakage of the main fragment ion (283). These compounds have been identified as 7-hydroxy-2-(2-phenylethyl)-6-{[(5*S*,6*S*,7*R*,8*S*)-5,6,7,8-tetrahydro-6,7,8-trihydroxy-4-oxo-2-(2-phenylethyl)-4H-chromen-5-yl]oxy}chromone isomers (AH15 isomers) [22], although it was not possible to distinguish between them because they showed the same fragmentation pattern. Twelve of them were detected in agarwood for the first time.

Peaks 93, 98, 103, 107, 110, and 115 gave a molecular ion at *m*/*z* 597 and were tentatively identified as AH12 isomers [22]. Peaks 94, 100, 102, 109, 116, 121, and 127, with the molecular formula C_34_H_28_O_8_, yielded the fragments at *m*/*z* 547, 529, 519, 283, 255, and 192. Where the product ion (*m*/*z* 192) corresponded to the loss of tetrahydroxy-5,6,7,8-tetrahydro-2-(2-phenylethyl)chromone group, the fragments at *m*/*z* 547, 529, and 519 matched the successive losses of H_2_O or CO. These compounds were tentatively identified as AH21 isomers, based on previously reported in *Aquilaria* species [23]. Six of them had never been reported in agarwood.

Peak 132, 16 Da (O) lighter than that of AH21 (565), gave a [M + H]^+^ ion at *m*/*z* 549 (C_34_H_28_O_7_). Accordingly, compound 132 was tentatively considered to be dehydroxy AH21. At the same time, Peaks 120, 125, 128, and 131 (*m*/*z* 579), 30 Da(OCH_2_) heavier than Peak 132 (549), presented the fragment ions at *m/z* 561, 543, and 121, resulting from the losses of two water molecules and the fragment of 121 [C_7_H_6_ + OCH_3_]^+^. Accordingly, these five compounds were tentatively considered to be 6-hydroxy-10-(4-methoxyphenethyl)-3-phenethyl-6,6a-dihydrodipyrano[3,2-a:2′,3′-i]dibenzo[b,e][1,4]dioxine-1,12(5H,14aH)-dione (methoxy AH21) isomers. All of them were potentially new compounds.

#### 2.1.4. Identification of Tri-2-(2-phenylethyl)chromones in Agarwood

**Figure 5:**
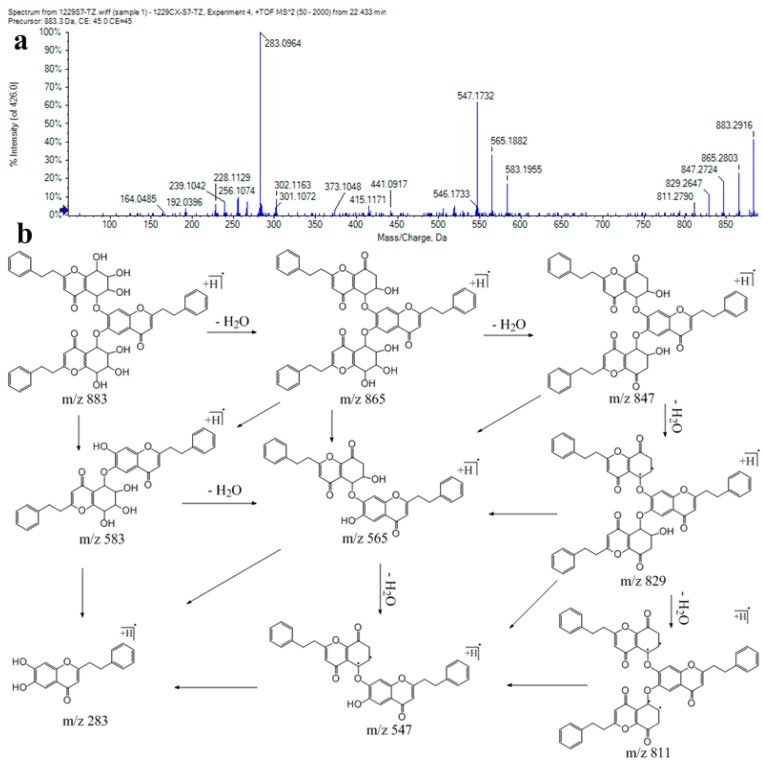
(**a**) MS/MS spectrum of compound 139; (**b**) proposed fragmentation pathway of compound 139.

Tri-2-(2-phenylethyl)chromones were minor in agarwood. Previous studies on agarwood revealed that only four tri-2-(2-phenylethyl)chromones were detected. The skeleton of tri-2-(2-phenylethyl)chromones is two 5,6,7,8-tetrahydro-2-(2-phenylethyl)chromones linked to a 2-(2-phenylethyl)chromone. Peaks 139, 140, and 141 showed molecular ions at *m*/*z* 883. In the MS/MS spectrum, fragment ions at *m*/*z* 583 and 283 corresponded to the loss of 5,6,7,8-tetrahydro-2-(2-phenylethyl)chromone from molecular ions. The significant ions at *m*/*z* 865, 847, 829, and 811, suggested the successive neutral losses of four waters, which resulted from the highly oxidized groups of two 5,6,7,8-tetrahydro-2-(2-phenylethyl)chromones. Other fragment ions were shown at *m*/*z* 565 and 547, resulting from the successive water losses from the fragment at *m*/*z* 583 (Figure 5). These compounds were tentatively identified as tri-2-(2-phenylethyl)chromone and its isomers [20,24].

### 2.2. Multivariate PCA and OPLS-DA Analysis of UPLC-MS Data

**Figure 6:**
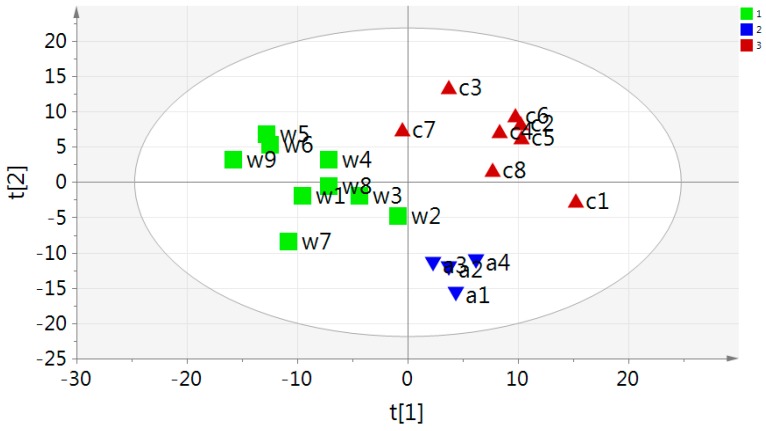
Principal component analysis (PCA) score plot of agarwood (group 1: wild agarwood; group 2 and 3: cultivated agarwood).

**Figure 7:**
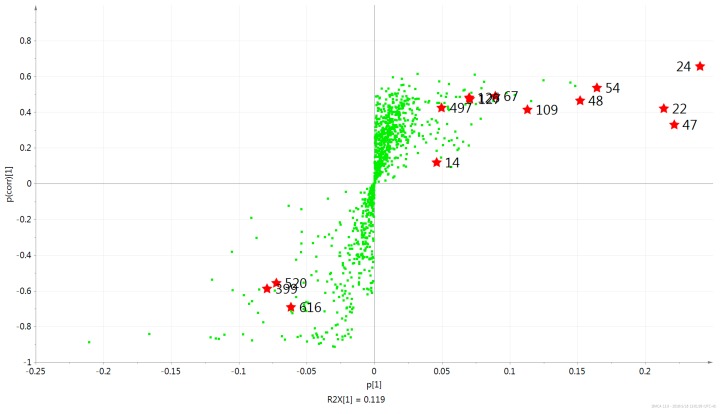
S-plot generated by orthogonal partial least squared discriminant analysis (OPLS-DA) for the metabolites in the wild and cultivated agarwood.

The unsupervised PCA score plot explained 71.0% of the total variance (R2). The agarwood samples were separated into a wild group (w) and two cultivated groups (a,c) (Figure 6). Next, all data sets of the wild and cultivated agarwood metabolite profiles were analyzed by supervised multivariate statistics and orthogonal partial least squared discriminant analysis (OPLS-DA). The S-plot easily visualized the variables that changed most significantly at the top or the bottom of the plot (Figure 7). The variables (peaks 13, 41, 42, 48, 50, 57, 58, 67, 69, 71, and 94) on the top showed the most upregulated metabolites in wild agarwood, and the ones (peaks 110, 113, and 123) on the bottom, the most downregulated. Among them, 14 metabolites were selected as biomarkers from the variables important in the projection (VIP) >1.0 in the statistical analysis. The variables were putatively assigned and presented in Table 3, which can serve as biomarkers between wild and cultivated agarwood. The main compounds were 6,7-dimethoxy-2-(2-phenylethyl)chromone, 6,8-dihydroxy-2-(2-phenylethyl)chromone, 6-methoxy-2-(2-phenylethyl)chromone, 6-methoxy-2-[2-(4-methoxyphenyl)ethyl]chromone, and others, as much different for the two types of agarwood samples. These biomarkers could significantly discriminate differences between groups. The results thus obtained are reliable and can be used for, for example, metabolic or biosynthetic pathway analysis.

## 3. Materials and Methods

### 3.1. Chemicals and Materials

Acetonitrile (Merck HPLC grade, Darmstadt, Germany) and formic acid (Sigma-Aldrich, MS Grade, St. Louis, MO, USA) were used. Ultrapure water was deionized and purified by the Milli-Q purification system (Millipore, Bedford, MA, USA). Other analytical grade reagents and chemicals were purchased from Guangzhou Chemical Reagent Factory (Guangzhou, China).

### 3.2. Plant Material

Twenty-one agarwood samples were selected and analyzed. All plantation-cultivated (via the whole-tree agarwood-Inducing technique (Agar-Wit, Zhongshan, China) and others) and wild-type agarwood (*A. sinensis*) was collected from Zhongshan, Chaozhou (Guangdong Province, China), and Haikou (Hainan Province, China) and identified by Xiaoping Lai (Guangzhou University of Chinese Medicine). Voucher specimens have been deposited at the New Drug Research & Development Center, Guangzhou University of Chinese Medicine.

### 3.3. Sample Preparation

Agarwood powder (0.2 g) was extracted with 10 mL of methanol by means of sonication at room temperature for 30 min. The extracts were concentrated under reduced pressure and then diluted to 10 μg·mL^−1^ with 70% aqueous acetonitrile. The solution was filtered through a 0.22-μm filter before UPLC-MS analysis.

### 3.4. UPLC Condition

UPLC analysis was performed on a Shimadzu Nexera LC-30A (Shimadzu, Kyoto, Japan), including an autosampler and a quaternary solvent delivery system. Separation was performed using an InertSustainSwift^TM^ C18 column (2.1 mm × 150 mm, 1.9 μm, Shimadzu-GL, Tokyo, Japan). The mobile phases were acetonitrile (A) and 0.1% formic acid–water (B). A gradient elution was used: 10% A at 0–3 min, 10%–30% A at 3–8 min, 30%–50% A at 8–25 min, 50%–100% A at 25–32 min, and 100% A at 32–35 min. The mobile phase was established at a flow rate of 0.3 mL·min^−1^, and the injection volume was 10 μL.

### 3.5. Mass Spectrometry

The quadrupole time-of-flight tandem mass spectrometry (QTOFMS) was performed using an AB-Sciex 5600 Triple TOF^TM^ mass spectrometer (Applied Biosystems/MDS Sciex, Foster City, CA, USA), coupled with DuoSpray^TM^ Ion Source (ESI and APCI, Foster City, CA, USA). The parameters were used as follows: ESI voltage, 4500 V; nebulizer gas, 55; auxiliary gas, 55; curtain gas, 35; turbo gas temperature, 500 °C; declustering potential, 100 V; collision energy, 10 eV. The samples were acquired with an IDA (Information-Dependent Acquisition) method, which can automatically select candidate ions for further product ion study. The TOF mass scan range was operated from *m*/*z* 100 to 2000, and the product ion scan range was *m*/*z* 50 to 2000. The collision energy (CE) was set from 45 eV. The MS calibration was set using the Calibrant Delivery System (CDS) by direct injection at a flow rate of 300 μL·min^−1^.

### 3.6. UPLC-MS Data Processing and Multivariate Analysis

Peak detection, alignment, and identification were processed using PeakView v2.0 and MarkerView v1.2.1 software (Applied Biosystems/MDS Sciex, Foster City, CA, USA). Statistical analyses with the 1066 × 21 matrix were carried out by SIMCA-P (version 13.0, Umetrics, Umea, Sweden). Unsupervised principal component analysis (PCA) was used for obtaining similarities or latent differences between groups. Orthogonal projection to latent structure discriminant analysis (OPLS-DA) was then implemented to detect maximum information from the data set and to distinguish the metabolites induced by different groups. Potential biomarkers were identified by analyzing the VIP and S-plot.

## 4. Conclusions

The UPLC–ESI/QTOF/MS-based metabolite-profiling approach enabled the tentative identification of 141 metabolites in agarwood extract on the basis of their accurate masses and MS/MS spectra in positive ion mode together with the reported data. The method applied combined the excellent separation effect of a small-particle-size C18-column (1.9 μm) and an IDA method; as such, the high resolution enabled the separation of a great number of isomers, with the high sensitivity, mass accuracy, and detections of the isotopic pattern combined with QTOF/MS for both molecular and fragment ions. We summarized the MS characterization of 2-(2-phenylethyl)chromones and identified these compounds for distinguishing wild from cultivated eaglewood. However, due to the fact that most of the compounds have the same molecular formula and similar fragment ions, some chromone isomers cannot be distinguished. This is the first time that a detailed study of these chromones has been carried out by UPLC-QTOF-MS analysis. At the same time, 2-(2-phenylethyl)chromones were used to distinguish wild from cultivated agarwood; 14 2-(2-phenylethyl)chromones were selected as biomarkers. The cultivars can be distinguished from the wild by their pattern. The present result was in line with some other findings that the ions of 267, 281, 283, 311, 313, 341, and 349 may be able to be differentiated into cultivated or wild [14,25] agarwood. It is interesting to note that there were 44 potentially new bi-2-(2-phenylethyl)chromones that had not been detected in previous work, and some of them can serve as biomarkers between wild and cultivated agarwood. The proposition for two types of agarwood needs to be further confirmed with a broader set of samples in future metabolic studies.

## Figures and Tables

**Table 1:** Retention time (RT) and mass spectral data of the compounds characterized in agarwood extract by ultra-performance liquid chromatography coupled with electrospray ionization mass spectrometry (UPLC–ESI-QTOF-MS) and MS/MS in positive mode (2-(2-phenylethyl)-chromones (unoxidized) and 5,6,7,8-tetrahydro-2-(2-phenylethyl)-chromones).

Peak	*RT* (min)	Formula	[M + H]^+^	Error ppm	MS/MS Fragments	Substituent Group		Proposed Compound
						A Ring	B Ring	
1	8.12	C_17_H_14_O_4_	283.0964	−0.2	192, 164, 91	OH, OH	−	6,8-dihydroxy-2-(2-phenylethyl)chromone (isomer 1)
2	10.6	C_18_H_16_O_6_	329.1011	−2.52	137	OH, OH	OH, OCH_3_	6,8-dihydroxy-2-[2-(3′-hydroxy-4′-methoxyphenyl)ethyl]chromone (isomer 1)
3	10.8	C_17_H_14_O_4_	283.0967	0.82	177, 107	OH	OH	6-hydroxy-2-[2-(4′-hydroxyphenyl)ethyl] chromone (isomer 2)
4	11.22	C_17_H_14_O_4_	283.0966	0.37	177, 107	OH	OH	6-hydroxy-2-[2-(4′-hydroxyphenyl)ethyl] chromone (isomer 3)
5	11.25	C_18_H_16_O_6_	329.1017	−0.86	137	OH, OH	OH, OCH_3_	6,8-dihydroxy-2-[2-(3′-hydroxy-4′-methoxyphenyl)ethyl]chromone (isomer 2)
6	12.08	C_17_H_14_O_4_	283.0963	−0.74	177, 107	OH	OH	6-hydroxy-2-[2-(4′-hydroxyphenyl)ethyl] chromone (isomer 4)
7	12.11	C_18_H_16_O_5_	313.1069	−0.37	207, 192, 107	OH, OCH_3_	OH	6-hydroxy-7-methoxy-2-[2-(4′-hydroxyphenyl)ethyl]chromone (isomer 1)
8	12.49	C_19_H_18_O_6_	343.1174	−0.76	207, 137	OCH_3_, OH	OCH_3_, OH	6-hydroxy-7-methoxy-2-(4′-hydroxy-3′-methoxyphenethyl) chromone (isomer 1)
9	12.71	C_17_H_14_O_4_	283.0963	−0.78	177, 107	OH	OH	6-hydroxy-2-[2-(4′-hydroxyphenyl)ethyl] chromone (isomer 5)
10	12.78	C_19_H_18_O_6_	343.1173	−0.8	206, 137	OCH_3_, OH	OCH_3_, OH	6-hydroxy-7-methoxy-2-(4′-hydroxy-3′-methoxyphenethyl) chromone (isomer 2)
11	12.94	C_19_H_18_O_6_	343.1173	−0.77	206, 137	OCH_3_, OH	OCH_3_, OH	6-hydroxy-7-methoxy-2-(4′-hydroxy-3′-methoxyphenethyl) chromone (isomer 3)
12	12.99	C_18_H_16_O_5_	313.1067	−0.98	177, 137	OH	OH, OCH_3_	6-hydroxy-2-[2-(4′-hydroxy-3′-methoxy phenyl)ethyl]chromone (isomer 1)
13	13.1	C_17_H_14_O_4_	283.0962	−0.87	192, 176, 91	OH, OH	−	6,8-dihydroxy-2-(2-phenylethyl)chromone (isomer 2)
14	13.45	C_18_H_16_O_5_	313.1067	−0.96	177, 137	OH	OH, OCH_3_	6-hydroxy-2-[2-(4′-hydroxy-3′-methoxy phenyl)ethyl]chromone (isomer 2)
15	13.67	C_18_H_16_O_5_	313.1071	0.17	121	OH, OH	OCH_3_	6,7-dihydroxy-2-[2-(4′-methoxyphenyl)ethyl]chromone (isomer 1)
16	13.69	C_19_H_18_O_5_	327.1224	−0.78	221, 177, 107	OCH_3_, OCH_3_	OH	qinanone g (isomer 1)
17	13.84	C_17_H_14_O_4_	283.0965	0.02	192, 164, 91	OH, OH	−	6,8-dihydroxy-2-(2-phenylethyl)chromone (isomer 3)
18	13.87	C_18_H_16_O_4_	297.112	−0.6	206, 167, 91	OH, OCH_3_	−	6-hydroxy-7-methoxy-2-[2-phenylethyl] chromone (isomer 1)
19	14.36	C_17_H_14_O_4_	283.0965	0.2	177, 107	OH	OH	6-hydroxy-2-[2-(4′-hydroxyphenyl)ethyl] chromone (isomer 1)
20	14.42	C_17_H_14_O_3_	267.1016	0.16	161, 107	−	OH	qinanone d (isomer 1)
21	14.5	C_19_H_18_O_5_	327.1228	0.4	221, 177, 107	OCH_3_, OCH_3_	OH	qinanone g (isomer 2)
22	14.74	C_20_H_20_O_6_	357.1333	0.06	221, 137	OCH_3_, OCH_3_	OH, OCH_3_	6,7-dimethoxy-2-[2-(3′-hydroxy-4′-methoxyphenyl)ethyl]chromone (isomer 1)
23	14.9	C_19_H_18_O_5_	327.1226	−0.3	236, 220, 207, 192, 91	OH, OCH_3_, OCH_3_	−	8-hydroxy-6,7-dimethoxy-2-(2-phenethyl)chromone
24	14.92	C_18_H_16_O_5_	313.1072	0.63	121	OH, OH	OCH_3_	6,7-dihydroxy-2-[2-(4′-methoxyphenyl)ethyl]chromone (isomer 2)
25	15.14	C_17_H_14_O_4_	283.0966	0.27	192, 153, 91	OH, OH	−	6,8-dihydroxy-2-(2-phenylethyl)chromone (isomer 4)
26	15.31	C_20_H_20_O_6_	357.133	−0.64	220, 137	OCH_3_, OCH_3_	OH, OCH_3_	6,7-dimethoxy-2-[2-(3′-hydroxy-4′-methoxyphenyl)ethyl]chromone (isomer 2)
27	15.58	C_17_H_14_O_3_	267.1014	−0.54	161, 107	−	OH	qinanone d (isomer 2)
28	15.87	C_17_H_14_O_3_	267.1013	−0.99	176, 91	OH	−	7-hydroxy-2-(2-phenylethyl)chromone (isomer 1)
29	15.87	C_18_H_16_O_4_	297.1119	−0.63	191, 107	OCH_3_	OH	6-methoxy-2-[2-(4′-hydroxyphenyl)ethyl] chromone (isomer 2)
30	16.37	C_19_H_18_O_5_	327.1227	0.02	220, 177, 107	OCH_3_, OCH_3_	OH	qinanone g (isomer 3)
31	17.05	C_18_H_16_O_4_	297.1121	−0.03	191, 107	OCH_3_	OH	6-methoxy-2-[2-(4′-hydroxyphenyl)ethyl] chromone (isomer 3)
32	17.15	C_17_H_14_O_3_	267.1013	−0.85	176, 91	OH	−	7-hydroxy-2-(2-phenylethyl)chromone (isomer 2)
33	17.32	C_19_H_18_O_5_	327.1228	0.35	191, 137	OCH_3_	OH, OCH_3_	6-methoxy-2-[2-(4′-hydroxy-3′-methoxyphenyl)ethyl]chromone (isomer 1)
34	17.55	C_18_H_16_O_4_	297.1121	−0.06	206, 167, 91	OH, OCH_3_	−	6-hydroxy-7-methoxy-2-(2-phenylethyl)chromone (isomer 2)
35	17.6	C_17_H_14_O_3_	267.1014	−0.46	161, 107	−	OH	qinanone d (isomer 3)
36	17.8	C_18_H_16_O_4_	297.112	−0.49	206, 167, 91	OH, OCH_3_	−	6-hydroxy-7-methoxy-2-(2-phenylethyl)chromone (isomer 4)
37	17.97	C_19_H_18_O_5_	327.1224	−0.92	191, 137	OCH_3_	OH, OCH_3_	6-methoxy-2-[2-(4′-hydroxy-3′-methoxyphenyl)ethyl]chromone (isomer 2)
38	18.37	C_19_H_18_O_6_	343.1174	−0.69	207, 137	OCH_3_, OH	OCH_3_, OH	6-hydroxy-7-methoxy-2-(4′-hydroxy-3′-methoxyphenethyl) chromone (isomer 4)
39	18.38	C_18_H_16_O_4_	297.1121	−0.15	176, 121	OH	OCH_3_	6-hydroxy-2-[2-(4′-methoxyphenyl)ethyl] chromone
40	18.38	C_19_H_18_O_5_	327.1225	−0.5	121	OH, OCH_3_	OCH_3_	7-hydroxy-6-methoxy-2-(4′-methoxyphenethyl)chromone (isomer 1)
41	18.45	C_18_H_16_O_5_	313.107	−0.09	206, 191, 107	OH, OCH_3_	OH	6-hydroxy-7-methoxy-2-[2-(4′-hydroxyphenyl)ethyl]chromone (isomer 2)
42	18.69	C_17_H_14_O_3_	267.1018	0.68	176, 91	OH	−	7-hydroxy-2-(2-phenylethyl)chromone (isomer 3)
43	18.99	C_19_H_18_O_6_	343.1173	−0.93	137	OCH_3_, OH	OCH_3_, OH	6-hydroxy-7-methoxy-2-(4′-hydroxy-3′-methoxyphenethyl) chromone (isomer 5)
44	19.13	C_18_H_16_O_4_	297.112	−0.51	191, 107	OCH_3_	OH	6-methoxy-2-[2-(4′-hydroxyphenyl)ethyl] chromone (isomer 1)
45	19.35	C_18_H_16_O_5_	313.1073	0.74	121	OH, OH	OCH_3_	6,7-dihydroxy-2-[2-(4′-methoxyphenyl)ethyl]chromone (isomer 3)
46	19.58	C_17_H_14_O_4_	283.0962	−0.96	192, 153, 91	OH, OH	−	6,8-dihydroxy-2-(2-phenylethyl)chromone (isomer 5)
47	20.35	C_18_H_16_O_5_	313.1067	−0.99	207, 192, 107	OH, OCH_3_	OH	6-hydroxy-7-methoxy-2-[2-(4′-hydroxyphenyl)ethyl]chromone (isomer 3)
48	20.63	C_20_H_20_O_5_	341.1386	0.83	220, 121	OCH_3_, OCH_3_	OCH_3_	6,7-dimethoxy-2-[2-(4′-methoxyphenyl)ethyl]chromone
49	20.91	C_18_H_16_O_5_	313.1076	1.6	207, 178, 91	OH, OH, OCH_3_	−	6,8-dihydroxy-7-methoxy-2-(2-phenylethyl)chromone
50	20.99	C_19_H_18_O_4_	311.1281	0.97	220, 205, 177, 91	OCH_3_, OCH_3_	−	6,7-dimethoxy-2-(2-phenylethyl)chromone
51	21.36	C_17_H_14_O_4_	283.0967	0.81	192, 164, 153, 91	OH, OH	−	6,8-dihydroxy-2-(2-phenylethyl)chromone (isomer 6)
52	22.51	C_18_H_16_O_3_	281.1173	0.25	160, 121	−	OCH_3_	2-[2-(4′-methoxyphenyl)ethyl]chromone
53	22.89	C_17_H_14_O_2_	251.1068	0.44	160, 91	−	−	2-(2-phenylethyl)chromone
54	23.41	C_18_H_16_O_3_	281.1172	−0.15	190, 91	OCH_3_	−	6-methoxy-2-(2-phenylethyl)chromone (isomer 1)
55	23.43	C_18_H_15_ClO_4_	331.0733	0.46	121	OH, Cl	OCH_3_	8-chloro-6-hydroxy-2-(4′-methoxyphenethyl)chromone
56	23.98	C_17_H_13_ClO_3_	301.0626	0.16	210, 170, 91	OH, Cl	−	8-chloro-6-hydroxy-2-(2-phenethyl) chromone
57	24.1	C_19_H_18_O_4_	311.128	0.69	190, 121	OCH_3_	OCH_3_	6-methoxy-2-[2-(4′-methoxyphenyl)ethyl]chromone
58	24.51	C_18_H_16_O_3_	281.1175	0.94	190, 91	OCH_3_	−	6-methoxy-2-(2-phenylethyl)chromone (isomer 2)
59	25.63	C_19_H_18_O_5_	327.1229	0.59	206, 121	OH, OCH_3_	OCH_3_	7-hydroxy-6-methoxy-2-(4′-methoxyphenethyl)chromone (isomer 2)
60	26.14	C_18_H_16_O_4_	297.1124	0.92	206, 167, 91	OH, OCH_3_	−	6-hydroxy-7-methoxy-2-(2-phenylethyl)chromone (isomer 3)
61	6.21	C_18_H_20_O_8_	365.1223	−2.08	137	OH, OH, OH, OH	OH, OCH_3_	aquilarone a
62	6.52	C_17_H_18_O_7_	335.1126	0.21	317, 299, 271, 243, 107	OH, OH, OH, OH	OH	aquilarone f (isomer 1)
63	7.23	C_17_H_18_O_7_	335.1126	0.09	317, 299, 271, 243, 107	OH, OH, OH, OH	OH	aquilarone f (isomer 2)
64	7.8	C_17_H_16_O_5_	301.107	−0.3	283, 255, 227, 192, 164, 91	OH, OH, –O–	−	2,3-dihydroxy-5-phenethyl-2,3-dihydro-1ah-oxireno[2,3-f]chromen-7(7bh)-one (isomer 1)
65	8.11	C_17_H_16_O_5_	301.1072	0.49	283,255,227,192,164,91	OH, OH, –O–	−	2,3-dihydroxy-5-phenethyl-2,3-dihydro-1ah-oxireno[2,3-f]chromen-7(7bh)-one (isomer 2)
66	8.12	C_17_H_18_O_6_	319.1178	0.6	301, 283, 255, 227, 164, 91	OH, OH, OH, OH	−	agarotetrol
67	8.37	C_18_H_20_O_7_	349.1282	−0.03	331, 313, 285, 121	OH, OH, OH, OH	OCH_3_	5,6,7,8-tetrahydroxy-2-(4-methoxyphenethyl)-5,6,7,8-tetrahydrochromone (isomer 1)
68	8.47	C_17_H_16_O_5_	301.1065	−1.83	283, 255, 227, 192, 164, 91	OH, OH, –O–	_	2,3-dihydroxy-5-phenethyl-2,3-dihydro-1ah-oxireno[2,3-f]chromen-7(7bh)-one (isomer 3)
69	8.65	C_17_H_18_O_6_	319.1178	0.61	301, 283, 255, 227, 164, 91	OH, OH, OH, OH	_	aquilarone b
70	8.66	C_17_H_16_O_5_	301.107	−0.04	283, 255, 227, 192, 164, 91	OH, OH, –O–	_	2,3-dihydroxy-5-phenethyl-2,3-dihydro-1ah-oxireno[2,3-f]chromen-7(7bh)-one (isomer 4)
71	8.85	C_18_H_20_O_7_	349.1279	−0.83	331, 313, 285, 121	OH, OH, OH, OH	OCH_3_	5,6,7,8-tetrahydroxy-2-(4-methoxyphenethyl)-5,6,7,8-tetrahydrochromone (isomer 2)
72	8.92	C_18_H_20_O_6_	333.1333	0.01	301, 283, 255, 227, 164, 91	OH, OH, OH, OCH_3_	_	5,6,7-trihydroxy-8-methoxy-5,6,7,8-tetrahydro-2-(2-phenylethyl)chromone (isomer 1)
73	8.95	C_17_H_18_O_5_	303.1225	−0.75	285, 267, 239, 211, 194, 176, 91	OH, OH, OH		5,6,7-trihydroxy-5,6,7,8-tetrahydro-2-(2-phenylethyl)chromone (isomer 1)
74	9.11	C_18_H_20_O_6_	333.133	−0.89	315, 121	OH, OH, OH	OCH_3_	5,6,7-trihydroxy-2-(4-methoxyphenethyl)-5,6,7,8-tetrahydrochromone (isomer 1)
75	9.61	C_18_H_20_O_6_	333.1334	0.44	301, 283, 255, 227, 164, 91	OH, OH, OH, OCH_3_	_	5,6,7-trihydroxy-8-methoxy-5,6,7,8-tetrahydro-2-(2-phenylethyl)chromone (isomer 2)
76	9.64	C_17_H_18_O_5_	303.1224	−0.86	285, 267, 239, 211, 194, 176, 91	OH, OH, OH	_	5,6,7-trihydroxy-5,6,7,8-tetrahydro-2-(2-phenylethyl)chromone (isomer 2)
77	10.22	C_17_H_18_O_5_	303.1224	−0.84	285, 267, 239, 211, 194, 176, 91	OH, OH, OH	_	5,6,7-trihydroxy-5,6,7,8-tetrahydro-2-(2-phenylethyl)chromone (isomer 3)
78	10.32	C_18_H_20_O_6_	333.1331	−0.58	315, 121	OH, OH, OH	OCH_3_	5,6,7-trihydroxy-2-(4-methoxyphenethyl)-5,6,7,8-tetrahydrochromone (isomer 1)
79	10.61	C_17_H_18_O_4_	287.1276	−0.7	269, 251, 178, 160, 91	OH, OH	_	6,7-dihydroxy-5,6,7,8-tetrahydro-2-(2-phenylethyl)chromone
80	10.77	C_17_H_18_O_5_	303.1221	−2.02	285, 267, 239, 211, 194, 176, 91	OH, OH, OH	_	5,6,7-trihydroxy-5,6,7,8-tetrahydro-2-(2-phenylethyl)chromone (isomer 4)
81	10.8	C_17_H_16_O_5_	301.107	−0.04	283, 255, 227, 192, 164, 91	OH, OH, –O–	_	2,3-dihydroxy-5-phenethyl-2,3-dihydro-1ah-oxireno[2,3-f]chromen-7(7bh)-one (isomer 5)
82	11.52	C_17_H_17_ClO_5_	337.0837	−0.08	319, 301, 283, 265, 192, 91	OH, OH, OH, Cl	_	8-chloro-5,6,7-trihydroxy-2-(2-phenylethyl)-5,6,7,8-tetrahydrochromone (isomer 1)
83	11.64	C_18_H_19_ClO_6_	367.0945	0.44	349, 121	OH, OH, OH, Cl	OCH_3_	8-chloro-5,6,7-trihydroxy-2-(4-methoxyphenethyl)-5,6,7,8-tetrahydrochromene (isomer 1)
84	11.68	C_17_H_17_ClO_5_	337.0835	−0.66	319, 301, 283, 265, 192, 91	OH, OH, OH, Cl	_	8-chloro-5,6,7-trihydroxy-2-(2-phenylethyl)-5,6,7,8-tetrahydrochromone (isomer 2)
85	12.01	C_17_H_16_O_5_	301.107	-0.32	283, 255, 227, 192, 164, 91	OH, OH, –O–	_	2,3-dihydroxy-5-phenethyl-2,3-dihydro-1ah-oxireno[2,3-f]chromen-7(7bh)-one (isomer 6)
86	12.82	C_18_H_19_ClO_6_	367.093	−3.41	349, 121	OH, OH, OH, Cl	OCH_3_	8-chloro-5,6,7-trihydroxy-2-(4-methoxyphenethyl)-5,6,7,8-tetrahydrochromene (isomer 2)
87	12.91	C_17_H_17_ClO_5_	337.0839	0.53	319, 301, 283, 265, 192, 9	OH, OH, OH, Cl	_	8-chloro-5,6,7-trihydroxy-2-(2-phenylethyl)-5,6,7,8-tetrahydrochromone (isomer 3)

**Table 2:** Retention time (RT) and mass spectral data of the compounds characterized in agarwood extract by UPLC–ESI-QTOF-MS and MS/MS in positive mode (bi-2-(2-phenylethyl)-chromones and tri-2-(2-phenylethyl)-chromones).

Peak	*RT* (min)	Formula	[M + H]^+^	Error ppm	MS/MS Fragments	Proposed Compound
88	16.68	C_34_H_30_O_9_	583.1965	0.37	565, 547, 355, 302, 283	AH15 (isomer 1) (B-type)
89	17.39	C_34_H_30_O_9_	583.1966	0.59	565, 547, 519, 459, 441, 283, 255, 228, 177	AH15 (isomer 2) (B-type)
90	18.3	C_34_H_30_O_9_	583.1951	−1.97	565, 547, 519, 415, 283, 192, 91	AH15 (isomer 3) (B-type)
91	18.85	C_34_H_30_O_8_	567.2014	0.14	549, 531, 503, 283, 267, 239, 91	AH13 (isomer 1) (B-type)
92	18.86	C_34_H_30_O_9_	583.1959	−0.68	565, 547, 519, 302, 283, 192, 91	AH15 (isomer 4) (B-type)
93	19.36	C_35_H_32_O_9_	597.2112	−1.21	579, 561, 297, 255, 121	AH12 (isomer 1) (B-type)
94	19.44	C_34_H_28_O_8_	565.1847	−1.81	547, 519, 283, 91	AH21 (isomer 1) (C-type)
95	19.47	C_34_H_30_O_9_	583.195	−2.14	547, 519, 283, 255, 192, 91	AH15 (isomer 5) (B-type)
96	19.52	C_34_H_30_O_8_	567.2005	−1.51	549, 531, 503, 283, 267, 239, 228, 192, 91	AH13 (isomer 2)(B-type)
97	19.84	C_34_H_30_O_9_	583.1954	−1.51	565, 547, 519, 301, 283, 192, 91	AH15 (isomer 6) (B-type)
98	19.95	C_35_H_32_O_9_	597.2115	−0.7	579, 561, 297, 283, 255, 206, 192, 121	AH12 (isomer 2) (B-type)
99	20.16	C_34_H_30_O_8_	567.2008	−0.89	549, 531, 513, 440, 283, 267, 255, 192, 176, 91	AH13 (isomer 3) (B-type)
100	20.42	C_34_H_28_O_8_	565.184	−3.02	547, 519, 301, 283, 192, 91	AH21 (isomer 2) (C-type)
101	20.77	C_34_H_30_O_9_	583.1959	−0.69	565, 547, 519, 267, 255, 91	AH15 (isomer 7) (B-type)
102	21.09	C_34_H_28_O_8_	565.1855	−0.42	547, 283, 192, 91	AH21 (isomer 3) (C-type)
103	21.48	C_35_H_32_O_9_	597.2115	−0.69	597, 561, 297, 255, 206	AH12 (isomer 3) (B-type)
104	21.61	C_34_H_30_O_9_	583.1962	−0.07	565, 547, 456, 283, 192, 91	AH15 (isomer 8) (B-type)
105	22.11	C_34_H_26_O_7_	547.1742	−1.76	529, 456, 282, 267, 91	hydroxy AH11 (isomer 1)(A-type)
106	22.19	C_34_H_30_O_9_	583.1957	−0.88	547, 519, 302, 283, 256, 91	AH15 (isomer 9) (B-type)
107	22.29	C_35_H_32_O_9_	597.2113	−1.08	579, 561, 297, 283, 267, 239, 206, 192, 121	AH12 (isomer 4) (B-type)
108	22.44	C_34_H_30_O_8_	567.2004	−1.69	549, 531, 513, 283, 255, 176, 91	AH13 (isomer 4) (B-type)
109	22.47	C_34_H_28_O_8_	565.1853	−0.73	547, 519, 283, 192, 91	AH21 (isomer 4) (C-type)
110	22.6	C_35_H_32_O_9_	597.2108	−1.9	579, 561, 297, 255, 239, 192, 121	AH12 (isomer 5) (B-type)
111	22.86	C_34_H_30_O_9_	583.1959	−0.58	547, 519, 302, 281, 192, 91	AH15 (isomer 10) (B-type)
112	23.13	C_34_H_26_O_7_	547.1746	−0.89	529, 456, 282, 267, 91	hydroxy AH11 (isomer 2 ) (A-type)
113	23.17	C_34_H_30_O_8_	567.2008	−0.96	549, 531, 440, 283, 267, 239, 192, 176, 91	AH13 (isomer 5) (B-type)
114	23.38	C_34_H_30_O_9_	583.1955	−1.24	547, 519, 302, 281, 267, 91	AH15 (isomer 11) (B-type)
115	23.53	C_35_H_32_O_9_	597.2108	−1.89	579, 561, 313, 269, 121	AH12 (isomer 6) (B-type)
116	23.55	C_34_H_28_O_8_	565.1851	−0.97	547, 519, 409, 283, 255, 192, 91	AH21 (isomer 5) (C-type)
117	23.98	C_34_H_30_O_8_	567.2008	−0.89	549, 531, 513, 440, 283, 267, 239, 91	AH13 (isomer 6) (B-type)
118	24.47	C_34_H_30_O_9_	583.196	−0.53	547, 415, 399, 303, 283	AH15 (isomer 12) (B-type)
119	25.15	C_34_H_30_O_9_	583.1963	0.1	547, 301, 283, 192, 91	AH15 (isomer 13) (B-type)
120	25.21	C_35_H_30_O_8_	579.201	−0.54	561, 543, 389, 371, 280	methoxy AH21 (isomer 1) (C-type)
121	25.25	C_34_H_28_O_8_	565.1853	−0.74	547, 519, 474, 456, 283, 91	AH21 (isomer 6) (C-type)
122	25.88	C_35_H_28_O_7_	561.1905	−0.58	470, 401, 121	methoxy AH11 (isomer 1) (A-type)
123	26.37	C_34_H_26_O_6_	531.1798	−0.79	440, 267, 91	AH11 (isomer 1) (A-type)
124	26.46	C_35_H_28_O_7_	561.1903	−0.94	470, 401, 121	methoxy AH11 (isomer 2)(A-type)
125	26.93	C_35_H_30_O_8_	579.2006	−1.36	561, 488, 470	methoxy AH21(isomer 2) (C-type)
126	27.06	C_34_H_26_O_7_	547.1747	−0.85	529, 456, 282, 267, 91	hydroxy AH11(isomer 3)(A-type)
127	27.37	C_34_H_28_O_8_	565.1844	−2.22	547, 529, 474, 439, 373, 283, 176, 91	AH21 (isomer 7) (C-type)
128	27.7	C_35_H_30_O_8_	579.2007	−1.13	561, 543, 529, 470, 283	methoxy AH21 (isomer 3) (C-type)
129	28.07	C_34_H_26_O_7_	547.1743	−1.5	529, 456, 282, 267, 91	hydroxy AH11(isomer 4)(A-type)
130	28.42	C_34_H_26_O_6_	531.1797	−0.9	440, 267, 91	AH11 (isomer 2) (A-type)
131	28.45	C_35_H_30_O_8_	579.201	−0.55	561, 543, 487, 458, 283, 121	methoxy AH21(isomer 4) (C-type)
132	28.64	C_34_H_28_O_7_	549.1903	−0.93	531, 458	dehydroxy AH21 (C-type)
133	28.7	C_34_H_29_O_7_Cl	585.1675	0.11	319, 301, 267, 176	6-((8-chloro-6,7-dihydroxy-4-oxo-2-phenethyl-5,6,7,8-tetrahydro-4H-chromen-5-yl)oxy)-2-phenethylchromone (B-type)
134	28.87	C_34_H_26_O_7_	547.1739	−2.24	529, 456, 282, 267, 91	hydroxy AH11(isomer 5) (A-type)
135	29.07	C_34_H_26_O_6_	531.1792	−1.84	440, 267, 91	AH11 (isomer 3) (A-type)
136	29.37	C_35_H_28_O_7_	561.1905	−0.55	470, 401, 121	methoxy AH11 (isomer 3) (A-type)
137	29.52	C_34_H_26_O_7_	547.1741	−1.79	529, 456, 282, 267, 91	hydroxy AH11(isomer 6) (A-type)
138	29.56	C_34_H_26_O_6_	531.1799	−0.65	440, 267, 91	AH11 (isomer 4) (A-type)
139	22.43	C_51_H_46_O_14_	883.2958	−0.29	865, 847, 829, 811, 583, 565, 547, 283	tri-2-(2-phenylethyl)chromone (isomer 1)
140	22.93	C_51_H_46_O_14_	883.2957	−0.4	865, 847, 829, 811, 583, 565, 547, 283	tri-2-(2-phenylethyl)chromone (isomer 2)
141	24.5	C_51_H_46_O_14_	883.296	−0.07	865, 847, 829, 811, 583, 565, 547, 301, 283	tri-2-(2-phenylethyl)chromone (isomer 3)

**Table 3:** Identified metabolites differing between wild and cultivated agarwood by UPLC-QTOF-MS.

Peak	Variable ID	VIP	Formula	Proposed Compound
50	47	7.70	C_19_H_18_O_4_	6,7-dimethoxy-2-(2-phenylethyl)chromone
13	24	7.50	C_17_H_14_O_4_	6,8-dihydroxy-2-(2-phenylethyl)chromone
58	22	6.83	C_17_H_14_O_3_	6-methoxy-2-(2-phenylethyl)chromone
57	48	4.81	C_19_H_18_O_4_	6-methoxy-2-[2-(4-methoxyphenyl)ethyl]chromone
41	54	4.52	C_18_H_16_O_5_	6-hydroxy-7-methoxy-2-[2-(4′-hydroxyphenyl)ethyl]chromone
48	109	4.01	C_20_H_20_O_5_	6,7-dimethoxy-2-[2-(4-methoxyphenyl)ethyl]chromone
42	14	3.06	C_17_H_14_O_3_	7-hydroxy-2-(2-phenylethyl)chromone
69	67	2.62	C_17_H_18_O_6_	Aquilarone B
123	399	2.13	C_34_H_26_O_6_	AH11
113	520	2.11	C_34_H_30_O_8_	AH13
71	127	1.94	C_18_H_20_O_7_	5,6,7,8-tetrahydroxy-2-(4-methoxyphenethyl)-5,6,7,8-tetrahydrochromone
67	126	1.91	C_18_H_20_O_7_	5,6,7,8-tetrahydroxy-2-(4-methoxyphenethyl)-5,6,7,8-tetrahydrochromone
110	616	1.68	C_35_H_32_O_9_	AH12
94	497	1.49	C_34_H_28_O_8_	AH21
